# *Escherichia coli* foster bladder cancer cell line progression via epithelial mesenchymal transition, stemness and metabolic reprogramming

**DOI:** 10.1038/s41598-020-74390-5

**Published:** 2020-10-22

**Authors:** Romaila Abd-El-Raouf, Salama A. Ouf, Mahmoud M. Gabr, Mahmoud M. Zakaria, Khaled F. El-Yasergy, Bedeir Ali-El-Dein

**Affiliations:** 1 Researches Department, Urology and Nephrology Center, Faculty of Medicine, Mansoura University, Mansoura, Egypt; 2 Botany and Microbiology Department, Faculty of Science, Cairo University, Giza, Egypt; 3 Urology Department, Urology and Nephrology Center, Faculty of Medicine, Mansoura University, Mansoura, Egypt

**Keywords:** Cancer, Microbiology, Medical research

## Abstract

Bacteria is recognized as opportunistic tumor inhabitant, giving rise to an environmental stress that may alter tumor microenvironment, which directs cancer behavior. In vitro infection of the T24 cell line with *E. coli* was performed to study the bacterial impact on bladder cancer cells. EMT markers were assessed using immunohistochemistry, western blot and RT-PCR. Stemness characteristics were monitored using RT-PCR. Furthermore, the metabolic reprograming was investigated by detection of ROS and metabolic markers. A significant (*p* ≤ 0.001) upregulation of vimentin as well as downregulation of CK19 transcription and protein levels was reported. A significant increase (*p* ≤ 0.001) in the expression level of stemness markers (CD44, NANOG, SOX2 and OCT4) was reported. ROS level was elevated, that led to a significant increase (*p* ≤ 0.001) in UCP2. This enhanced a significant increase (*p* ≤ 0.001) in PDK1 to significantly downregulate PDH (*p* ≤ 0.001) in order to block oxidative phosphorylation in favor of glycolysis. This resulted in a significant decrease (*p* ≤ 0.001) of AMPK, and a significant elevation (*p* ≤ 0.001) of MCT1 to export the produced lactate to extracellular matrix. Thus, bacteria may induce alteration to the heterogonous tumor cell population through EMT, CSCs and metabolic reprogramming, which may improve cancer cell ability to migrate and self-renew.

## Introduction

Bladder cancer (BC) is one of the most common cancers as the final American Cancer Society report estimated it as the fourth common cancer in men at 2020 in the United States^1^. BC is a major public health problem as the primary tumor of BC can be successfully controlled, but the subsequent growth of dormant metastatic residues is a vital obstacle to eradicate the disease^2^. Therefore, elucidation of the progression mechanisms is critical for BC curbing. Tumor metastasis has been attributed in part to three hallmark programs a. epithelial mesenchymal transition (EMT) b. cancer stem cells (CSCs) and c. metabolic reprograming^3,4^, which lead to phenotypic and metabolic changes reinforcing tumor progression^3,5^.

Epithelial cells may undergo EMT, which is a physiological phenotypic shift that endows epithelial cancer cell with mesenchymal features and downs epithelial characteristics, in order to acquire static cancer cell the ability of migration and invasion. This process occurs naturally during wound healing and embryogenesis, while accumulating evidence has pointed it out as a central driver of cancer metastasis^3,6^. EMT is orchestrated by transcriptional factors cohort which lead to loss of the expression of certain epithelial adhesion and structural molecules (e.g. cytokeratin 19 (CK19) and E cadherin), while increase the expression of mesenchymal cell molecules (e.g. vimentin)^4^. Some factors trigger EMT induction, such as extracellular factors (e.g. TGF-β, EGF, and HGF) in addition to tumor hypoxia inducible factors (e.g., HIF-1α and HIF-2α)^6^. Interestingly, EMT program activation has been connected to generating cancer stem-like cells and metabolic reprograming^7^.

The second efficient player in cancer progression is cancer stem cells (CSCs)^8^. Basically, tumor is portrayed as a homogenous cell population at the initial stages of the tumorigenesis, while during development, various subpopulations generate and cause tumor heterogeneity^9^. Thus, CSC is a small tumor subpopulation with a unique gene signature, which may has the ability to distinguish between papillary and invasive bladder cancer^10^. CSCs possess high plasticity and self-renewal capability that are crucial to expansion and differentiation into all tumor cell types, thus resembling the function of normal stem cells^10^. Cells with CSCs features possess higher resistance to chemotherapies and cause tumor dormancy^9^.

In fact, cancer cells support their survival and metastasis via their capability to alter cell metabolism to an anomalous state^9,11^. In normal cells, under normoxia, the predominant fate of glycolytic pyruvate is mitochondrial oxidation to carbon dioxide, which is called oxidative phosphorylation (OXPHOS)^5^. The other fate of pyruvate is glycolysis (lactate generation), which is observed in normal cells under hypoxia or rapid proliferation. The metabolic reprograming from OXPHOS to glycolysis is preferred in cancer cells, what is called Warburg effect. Metabolic reprograming helps dividing cancer cells to meet their basic needs of rapid ATP for synthesis of new biomass while keeping adequate ATP for cell survival, and maintaining appropriate redox status.

Bacteria is regarded as tumor inhabitant due to the poor immune activity within tumors. Besides that, bacteria may take the advantage of oxygen tension and permissive carbon sources, therefore the tumor microenvironment (TM) becomes a potential refuge for bacteria^12,13^. It is noteworthy that the relation between cancer and bacteria is intertwined. Bacteria could have anti-tumor effect, as they may deplete nutrients that are required for metabolism of tumor cells^14^. For instance, *Bifidobacteria* and *Salmonella* can invade solid tumors resulting in either retardation of neoplasm growth, or complete clearance of tumor^14^. Conversely, bacteria may play a role in carcinogenesis, for example chronic urinary tract infection (UTI) is significantly associated with increasing BC risk^15^. Furthermore, intratumor bacteria may act as cancer allies and support its development, for instance, acute bacterial infection increases metastasis to the lung^16^ and *Helicobacter pylori* has implicated in gastric cancer development^17^.

Understanding how could bacteria influence cancer cells may help to elucidate cancer progression and provide useful therapeutic targets for treatment and prevention of metastatic cancer. Therefore, the aim of this work is to evaluate the role played by bacterial stress on cancer progression by in vitro investigating the potential of *E. coli* in driving the three cancer hallmarks EMT, CSCs and metabolic reprograming.

## Materials and methods

### Bacterial isolation and identification

*Escherichia coli* strain was isolated from urine sample of bladder cancer patient during our previous work^18^. Urine sample collection was done according to ethical committee approval from Mansoura University (RP/41). The isolated bacterial species was excellently identified using automated VITEK 2 (Biomérieux, Marcy I' Etoile, France) according to manufactory instructions with the bionumber 045610450524610. The biochemical identification and antibiotic susceptibility details for this isolate are shown in Supplementary Information Tables S1 and S2.

### T24 culture and in vitro infection

T24 cell line, which was isolated from human uroepithelial carcinoma, was obtained from American Type Culture Collection (ATCC). Cells were cultured in Dulbecco’s Modified Eagle’s Medium (DMEM, Sigma, St. Louis, Missouri, USA) supplemented with 10% fetal bovine serum (FBS) at 37 °C in 5% CO_2_ and humidified atmosphere until reach 80% confluency. The cells were cultured in serum free media overnight, then in vitro infection with *E. coli* at multiplicity of infection (MOI) 20:1 per cell was performed. Then, two hours after infection, the cells were washed with 100 mg/ml Gentamycin containing phosphate buffered saline (PBS), followed by incubation in 10 mg/ml Gentamycin containing medium (DMEM, 10% FBS). Control T24 cells, were cultured for four days without bacterial infection. The infected and noninfected T24 cells were imaged before infection and after four days of infection, with phase contrast microscope (Olympus CKX41, Tokyo, Japan) to assess any morphological changes resembling EMT process. This experiment was performed three times.

### Immunohistochemistry (IHC)

The infected and control T24 cells were trypsinised and collected for the subsequent IHC assay. Cells were resuspended in PBS at a final concentration of 5 × 10^4^ cells/ml. The cytospin method was performed to fix the cells on the microscope slide. After that, cells were fixed for 15 min in 4% paraformaldehyde, followed by permeabilization with 0.25% Triton X-100 in PBS for ten minutes. To block non-specific binding, cells were incubated with bovine serum albumin (BSA) in (PBS) for half an hour. The slides were immunostained with a mouse anti-human CK19 monoclonal antibody (1:100, Genemed, USA), and a mouse anti-human vimentin monoclonal antibody (1:500, Sigma Aldrich, USA). The detection system (Power-StainTM 1.0 Poly HRP DAB Kit for Mouse + Rabbit, USA) was utilized to locate where the primary antibody is bound. The staining location was subsequently examined under the light microscope (CX31RTSF; Olympus, Tokyo, Japan). The mean percentage of CK19 and vimentin positively stained cells was generated from three separated experiments by counting the number of positive cells and negative cells in five different fields, randomly taken from each sample.

### Western blotting analysis

The infected and non-infected T24 monolayer cells (about 5 × 10^6^ cell for each) were washed using cold PBS. The cells were harvested in 1 ml PBS using cell scraper. Total protein was extracted from the cells using Ready Prep TM protein extraction kit (Bio-Rad, USA). Protein concentration was assessed using Bradford Protein Assay Kit (Bio basic, Canada). Equal amounts of protein (20 μg) from each sample was resolved by 10% SDS–PAGE, and the separated proteins were transferred to a 0.22 µm polyvinylidene fluoride membrane (Millipore, Bedford). After that, the membranes were incubated overnight at 4 °C with a mouse anti-human CK19 monoclonal antibody (1:300, Santa Cruz Biotechnology, USA), a mouse anti-human vimentin monoclonal antibody (1:300, Santa Cruz Biotechnology, USA), and a mouse anti-human β-actin monoclonal antibody (1:500, Santa Cruz Biotechnology, USA) in blocking buffer. HRP-conjugated secondary antibody (Universal Quick anti-mouse IgG, Novus Biologicals, USA) solution was applied against the blotted target protein for one hour at room temperature. The chemiluminescent substrate (ClarityTM Western ECL substrate Bio-Rad, USA) was applied to the blot, and the chemiluminescent signals was captured using a CCD camera-based imager.

### Gene expression analysis

T24 cells were seeded into 6-well plates with density (30 × 10^4^/well) and in vitro infection was performed as previously describes but with different MOIs (1:1, 1:5, 1:15 and 1:20). After that, cells were washed and incubated in 10 mg/ml Gentamycin containing medium (DMEM, 10% FBS). The cells were trypsinized and collected after two and four days of infection. Control T24 cells, were cultured and collected after two days and four days without bacterial infection.

### RNA extraction and complementary DNA synthesis

Total RNA was extracted from all samples using RNeasy Plus Mini Kit (Qiagen GmbH, Hilden, Germany). The extracted RNA was visualized using gel electrophoresis and was quantified using Nanodrop 2000 (Thermo Scientific, Wilminton, DE). cDNA was synthesized from 1 µg of RNA using the High Capacity cDNA Reverse Transcription Kit, (Thermo Fisher Scientific, USA) according to manufacturer’s instructions. cDNA samples were preserved at − 20 °C for further real time PCR analysis.

### PCR primers

Oligonucleotide primers for genes related to the three cancer hallmarks: (1) (EMT (CK19 and vimentin), (2) CSCs, which were purchased from Vivantis (Selangor, Malaysia), are listed in Table 1. (CD44, SOX2 (also known as SRY), NANOG homebox (NANOG), and POU class 5 homeobox 1 (OCT4) (also known as POU5F1)), (3) metabolic reprograming (Uncoupling protein 2 (UCP2), pyruvate dehydrogenase kinase 1 (PDK1), pyruvate dehyrogenase phosphatase( PDH, also known as PDP1), protein kinase AMP-activated (AMPK, also known as PRKAA2) and malonyl-CoA-acyl carrier protein transacylase (MCT1)) and for the housekeeping gene (GAPDH) were designed with the assistance of NCBI. The sequence of the primers which were purchased from Vivantis (Selangor, Malaysia) are listed in Table 1.

**Table 1:** Primer sequences used in RT-PCR.

Gene	Sequence	Annealing temp	Acc. no.	Target sequence
GAPDH	F: TCTTTTGCGTCGCCAGCC	60	NM_002046.5	178
	R: ACATGTAAACCATGTAGTTGAGGTC			
**EMT markers**				
CK19	F: TGCGGGACAAGATTCTTGGT	58	NM_002276.5	102
	R: TCTCAAACTTGGTTCGGAAGTCA			
Vimentin	F: CGGGAGAAATTGCAGGAGGA	60	NM_003380.3	105
	R: AAGGTCAAGACGTGCCAGAG			
**CSCs markers**				
CD44	F: TTTGCATTGCAGTCAACAGTC	60	XM_005253231.3	233
	R: TTACACCCCAATCTTCATGTCCAC			
NANONG	F: GAAGGCCTCAGCACCTACCT	60	NM_024865.3	95
	R: GGTTGCTCCACATTGGAAGGTT			
SOX2	F: GGATAAGTACACGCTGCCCG	60	NM_003106.3	111
	R: CTGTCCATGCGCTGGTTCAC			
OCT4	F: TGCCAAGCTCCTGAAGCAGA	60	NM_002701.5	100
	R: CGTTTGGCTGAATACCTTCCCAAA			
**Metabolic reprograming markers**				
UCP2	F: TGCGGCTCGGACACATAGTA	60	NM_003355.2	76
	R: CCGATCCCCTTGGTTTTCCA			
PDH	F: TTCTCAGAAGCCGGCAAGCA	60	NM_001173455.1	98
	R: CTTCCAGCCGGTGAAGGTCA			
PDK1	F: TCAACTGCACCAAGACCTCGT	60	XM_011511345.3	60
	R: ACCAAAACCAGCCAGAGGGA			
AMPK1	F: GCCATGCGCAGACTCAGTTC	60	NM_006251.5	83
	R: TAGTGGCCGATCTTCACCCG			
MCT1	F: GTTGGTGGCTGCTTGTCAGG	60	NM_003051.3	107
	R: AAGGCAAGCCCAAGACCTCC			

### PCR amplification and real-time quantitation

cDNA templates were amplified using PCR thermal cycler (CFX96 Real-Time System, Bio-Rad, USA). The amplification reaction contained a 20-μL total volume mixture, which consists of 10 μL of 2 × Maxima SYBR Green master mix (Thermo Fisher Scientific, USA), 1.5 μL of cDNA template, 2 μL of 10 pmol gene primer and 6.5 μL of nuclease-free water. The specificity of the PCR products was evaluated by melting curve analysis. GAPDH gene was used to normalize samples, and the fold changes of gene expression were calculated.

### Reactive oxygen species detection

After the cultures of infected and control T24 cells were trypsinized, the live cells were counted using the hemocytometer. The non-infected T24 cells were used as the negative control and uninfected T24 cells with H_2_O_2_ were regarded as the positive control. 0.5 × 10^6^ cell of each control and infected cells were taken and incubated separately in covered-falcon tubes with pre-warmed media containing 5 μM of 2′,7′-dichlorofluorescein diacetate (DCFH-DA, Sigma-Aldrich, St. Louis, MO, USA) for 30 min at 37 °C. In fact, DCFH-DA has the ability to cross the live cell membrane, undergo intracellular de-esterification and turn into highly fluorescent 2′,7′-dichlorofluorescein (DCF) upon oxidation. After that, cells were pelleted, extensively washed and re-suspended in pre-warm PBS, then were seeded into a 96 well dark-sided plate with a density of 5 × 10^4^ cell/well to be measured. The generation of intracellular ROS was evaluated by measuring the fluorescent intensity of resulted DCF using fluorescence microplate reader (Tecan's Spark 20 M multimode microplate reader, Switzerland) with a 480 nm excitation filter and a 535 nm emission filter. The mean fluorescence intensity (MFI ± SE) was calculated from three independent experiments. Moreover, fluorescent microscopy (Olympus CKX41) was used to immediate visualize the fluorescent intensity and the generated images was analyzed using ImageJ software.

### Statistical methods

Statistical analysis was performed using the SPSS 17 (Chicago, IL, USA). The values were expressed as mean ± SD for the samples generated from three separated experiments. To test the data distribution, the Kolmogorov–Smirnov test (K–S test) was performed. To evaluate the significance of changes due to *E. coli* infection regardless both MOI and infection duration, the independent sample T test was performed. While, the one-way ANOVA test was carried out to assess statistical significance among variables with respect to MOI and duration of infection, separately. Then, to compare the control with experimental data sets, the post-hoc Dunnett's test was conducted. In addition, the correlation between expressions of CK19 and vimentin was tested by the Pearson test (r). Statistical significances were considered if *p* values were less than 0.01.

### Ethical approval and consent to participate

Urine sample used for bacterial isolation was collected according to ethical committee approval from Mansoura Faculty of Medicine Institutional Research Board (MFM-IRB), Egypt (Approval no: RP/41). All procedures performed in studies involving human participants were in accordance with the ethical standards of the institutional and/or national research committee and with the 1964 Helsinki declaration and its later amendments or comparable ethical standards.

## Results

- Assessment of EMT features

**Figure 1:**
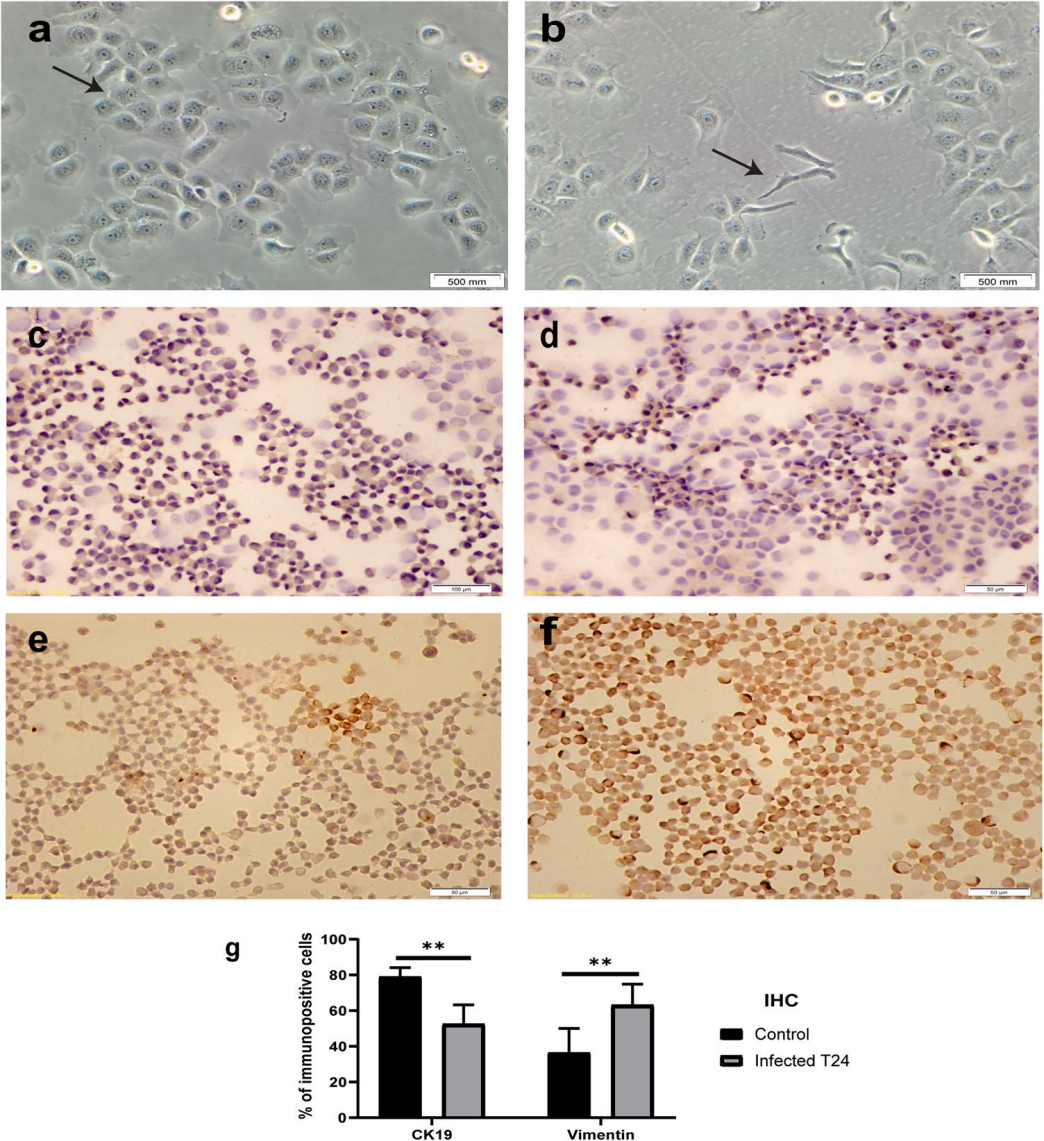
Shows the effect of *E. coli* infection on EMT: (**a**, **b)** show the phase contrast microscope images for T24 cell line before and after 4 days of infection, respectively. The arrows point out the change in morphology resembling EMT, resulted from bacterial infection. (**c**, **d**) show the IHC microscopic images for CK19 in T24 cell line before and after 4 days of infection. (**e**, **f**) show the IHC images for vimentin in T24 cell line before and after 4 days of infection. (**g**) shows the quantification data for CK19 and vimentin IHC images. The mean percentage of CK19 expression level decreased from 79 to 51% and the mean percentage of vimentin expression level increased from 36 to 65%. **Express the significance (*p* ≤ 0.001) obtained by independent t test, n = 15.

Infected T24 cultures possessed a particular morphology with elongated spindle-shape and shortage of intercellular contacts. While uninfected T24 cells possessed more epithelial morphology and a clear cell–cell adhesion, with polygonal shape and more regular dimensions. This newly rearranged cell shape is resembling the EMT phenotype as illustrated in Fig. 1(a, b).

Consistent with these findings, immunohistochemistry analysis illustrated an increase in the mean percentage of vimentin positive stained T24 cells following four days of bacterial exposure from 36 to 65% (Fig. 1c, d). This change was also accompanied by a decrease of CK19 expression of stimulated cells from 79 to 51% when compared with controls (Fig. 1e, f). The quantification data for the immunopositive cells was illustrated in Fig. 1(g).

**Figure 2:**
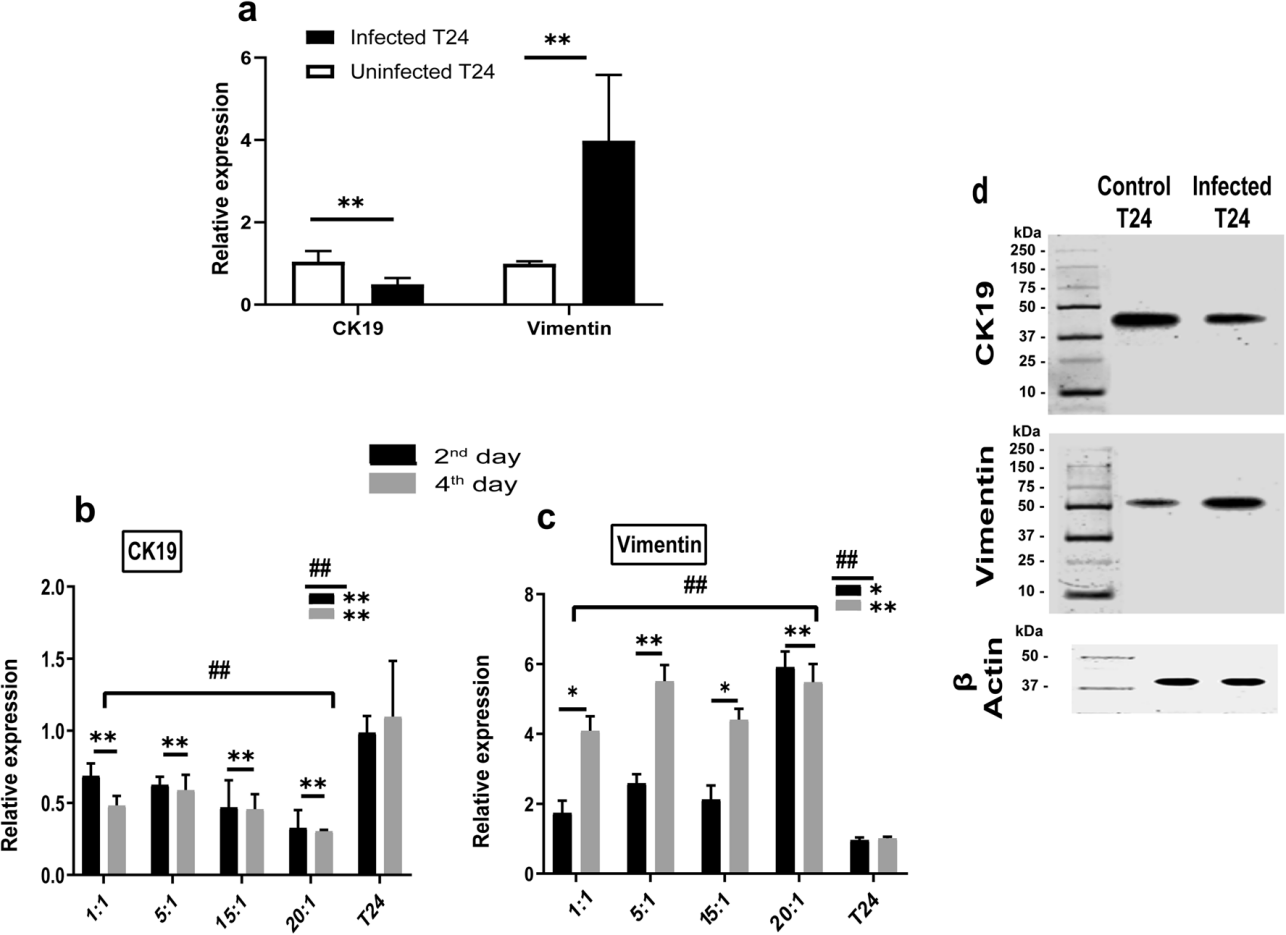
Shows the effect of *E. coli* infection on EMT-related genes and proteins: (**a**) shows the pattern of CK19 and vimentin expression in T24 cell line before and after infection. **Express the significance (*p* ≤ 0.001) obtained by independent t test. Infected T24 n = 24, control T24 n = 6. (**b**, **c**) show the CK19 and vimentin expression in T24 cell line after *E. coli* infection at different MOI and durations, compared to uninfected T24 cells. #, ## Express the significant difference (*p* ≤ 0.01) and (*p* ≤ 0.001) of gene expression with respect to the infection duration and MOI respectively, obtained by one-way ANOVA. *, **Refers to the significant difference (*p* ≤ 0.01) and (*p* ≤ 0.001) obtained by Post Hoc Dunnett t (2-sided) for each MOI and each duration compared against the control T24 cells. 2nd day n = 12, 4th day n = 12, each MOI n = 6 and control n = 6. (**d**) shows the western blot images for CK19 and vimentin in T24 cell line before and after infection. The full-length blots are presented in Supplementary Information Figure S2.

Moreover, RT-PCR analysis demonstrated that the T24 cells exposure to bacterial resulted in gene expression profile representative to EMT (Fig. 2). Overall, the infected T24 cells had the lowest expression level of CK19 (0.5 ± 0.2 folds) and the highest expression levels of vimentin (4 ± 1.5 folds), relative to control. Regarding to bacterial stimulation, the CK19 transcription levels decreased significantly at *p* ≤ 0.001, while the vimentin transcription levels increased significantly at *p* ≤ 0.001, when compared with the corresponding levels in uninfected T24 cells (Fig. 2a). At the end of the 4th day of infection, the CK19 expression levels reduced to (0.5 ± 0.09) folds while vimentin expression levels increased up to (4 ± 0.6) folds. During the four days following the infection, the CK19 and the vimentin transcription levels have changed significantly at *p* ≤ 0.001, with respect to both MOI and duration of infection, separately (Fig. 2b, c). Post Hoc Dunnett t (2-sided) test was performed to confirm the significance at each MOI and duration separately compared to control (Fig. 2b, c). A significant correlation between changes in both genes due to bacterial stimulation was reported (r: − 0.89, f: *p* ≤ 0.001). Furthermore, western blot analysis emphasizes that bacterial infection led to decrease in CK19 protein level and increase in vimentin protein level as shown in Fig. 2(d).

- Assessment of CSCs associated genes

**Figure 3:**
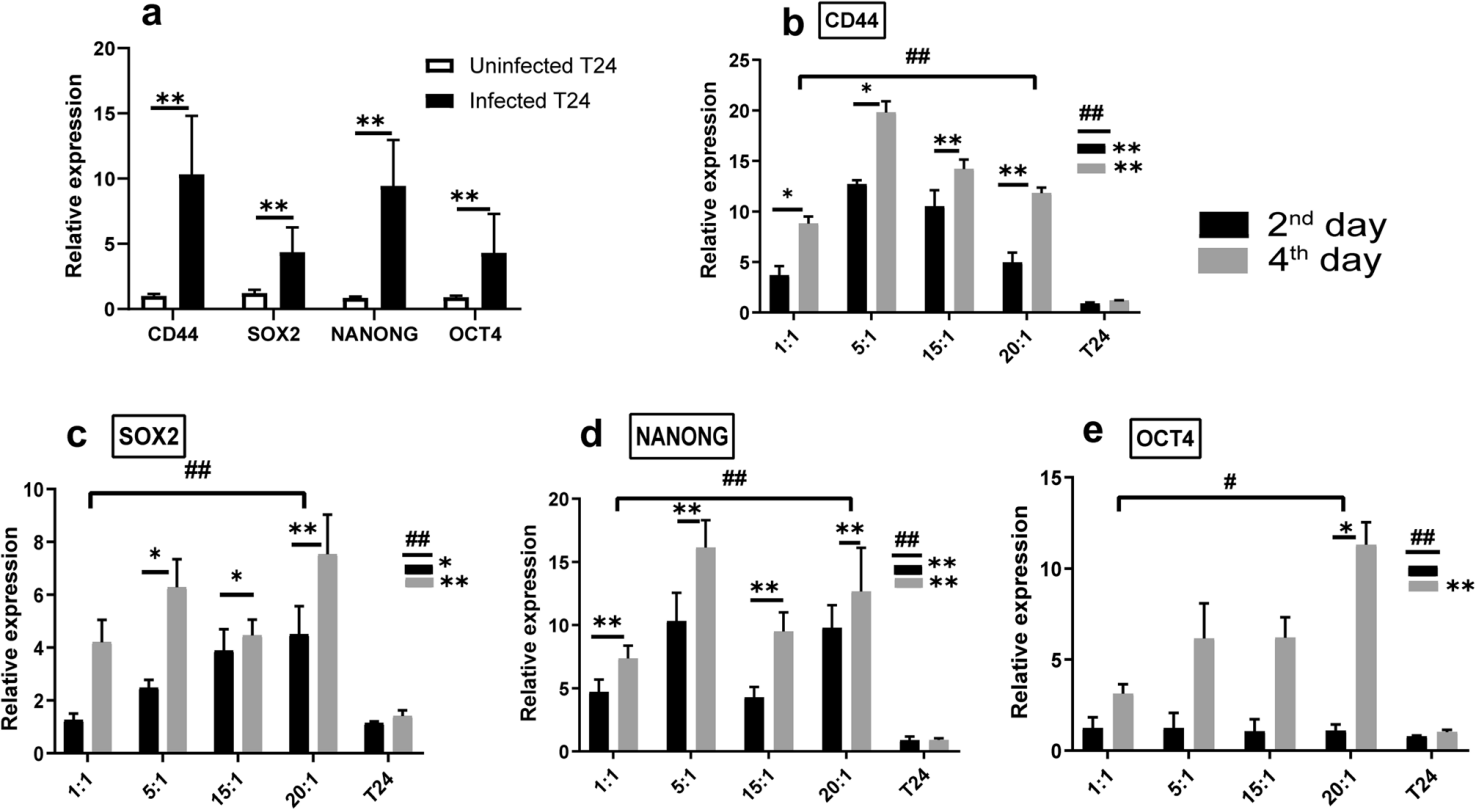
Shows the effect of *E. coli* infection on relative gene expression of stemness-related genes: (**a**) shows the pattern of CD44, SOX2, NANOG and OCT4 expression in T24 cell line before and after *E. coli* infection. **Express the significance (*p* ≤ 0.001) obtained by independent t test. Infected T24 n = 24, control T24 n = 6. (**b**, **c**, **d**, **e**) show the CD44, SOX2, NANOG and OCT4 expression in T24 cell line after *E. coli* infection at different MOI and durations, compared to uninfected T24 cells. #, ## Express the significant difference (*p* ≤ 0.01) and (*p* ≤ 0.001) of gene expression with respect to the infection duration and MOI respectively, obtained by one-way ANOVA. *, **Refers to significant difference (*p* ≤ 0.01) and (*p* ≤ 0.001) obtained by Post Hoc Dunnett t (2-sided) for each MOI and each duration compared against the control T24 cells. 2nd day n = 12, 4th day n = 12, each MOI n = 6 and control n = 6.

PCR analysis has pointed out significant changes to the four-stemness factors (CD44, SOX2, NANOG and OCT4) gene expression as shown in Fig. 3. Overall, the infected T24 cells recorded the highest mean expression levels of CD44 (10 ± 4 folds), SOX2 (4.3 ± 1.8 folds), NANOG (9.4 ± 3.5 folds) and OCT4 (4.3 ± 2.9 folds) relative to control (Fig. 3a).

Regarding to bacterial stimulation, CD44, SOX2, NANOG, and OCT4 transcript levels increased significantly at *p* ≤ 0.001, when compared with the corresponding levels in control T24 cells (Fig. 3a). At 4th day after *E. coli* stimulation, the highest expression levels were detected, with mean fold increase about (12.6 ± 3.7, 5.6 ± 1.5, 11 ± 3 and 6 ± 2.3 folds) for CD44, SOX2, NANOG, and OCT4, respectively relative to controls.

During the four days following the infection, the CD44, SOX2, NANOG, and OCT4 levels have changed significantly at *p* ≤ 0.001 with respect to duration of infection. Regarding MOI, all stemness markers have changed significantly at *p* ≤ 0.001, except OCT4, as it changed significantly at *p* ≤ 0.01. Post Hoc Dunnett t (2-sided) test was performed to confirm the significance at each MOI and duration separately compared to control (Fig. 3b–e). These finding support the induction of cancer stem cell like phenotype by *E. coli* infection.

- Assessment of metabolic reprograming-associated indicators

**Figure 4:**
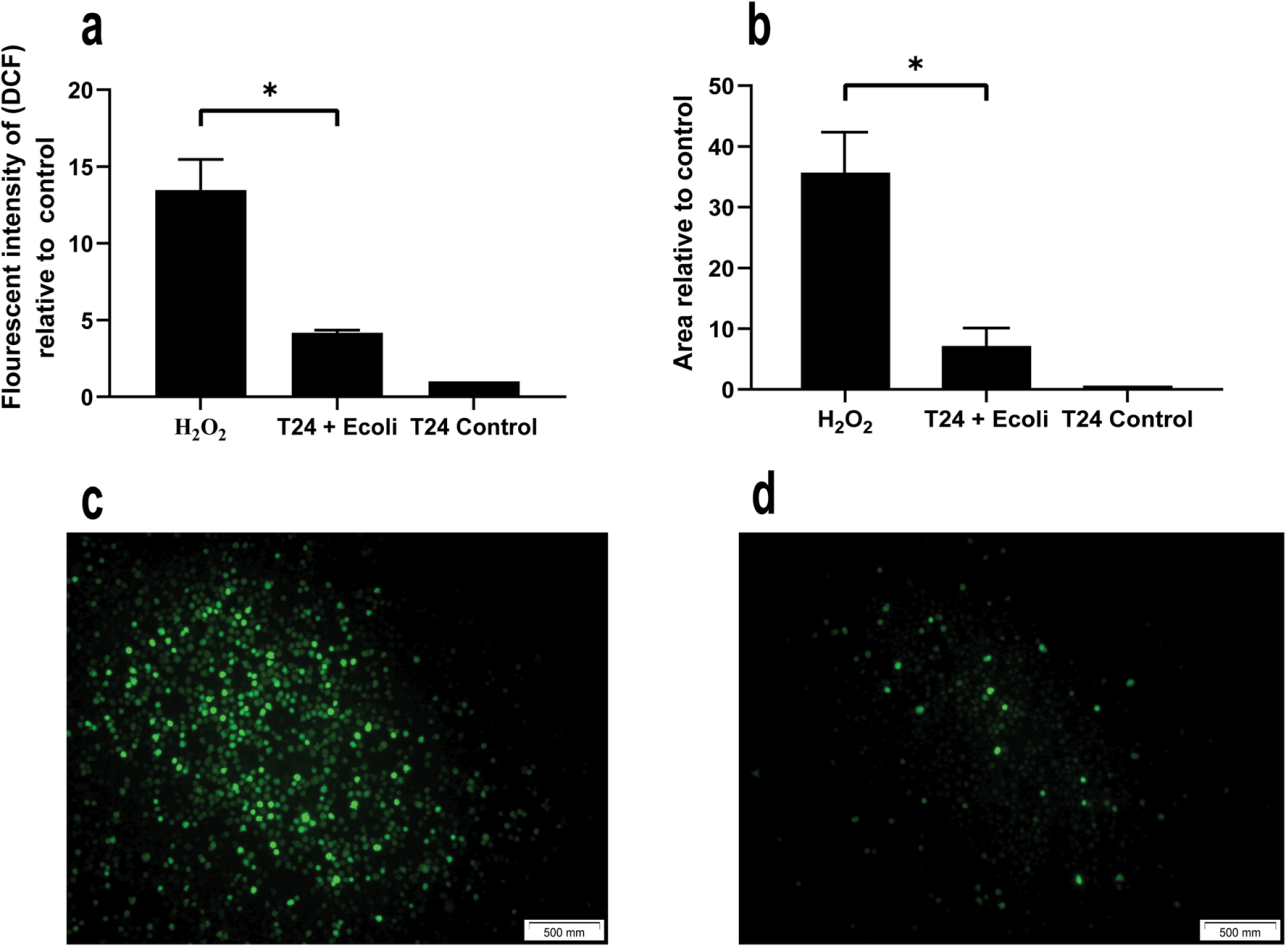
Effect of bacterial infection on ROS in T24 cells after 4 days of bacterial infection: (**a**) shows the amount of intracellular ROS relative to control, detected using fluorescent microplate reader TECAN. *Express the significance at (*p* ≤ 0.01) obtained by independent t test, n = 3. (**b**) Shows the amount of intracellular ROS relative to control, detected using image analysis for the fluorescent microscope images using ImageJ software. *Express the significance at (*p* ≤ 0.01) obtained by independent t test, n = 3. (**c**, **d**) show the fluorescent microscope images for ROS in T24 cell line before and after 4 days of bacterial infection, respectively.

We hypothesized that the intracellular production of ROS of infected T24 cells would be correlated to metabolic reprograming. The assessment of DCF was performed to detect the change in ROS production caused by bacterial infection. The Tecan intracellular DCF fluorescent quantitative analysis relative to control is shown in Fig. 4(a), which emphasized the overproduction of ROS due to infection. The fluorescent microscopic images for infected and non-infected T24 were analyzed using image J software and the changes in the fluorescent area were illustrated in Fig. 4(b). The fluorescent microscopic images were illustrated in Fig. 4(c, d), respectively.

**Figure 5:**
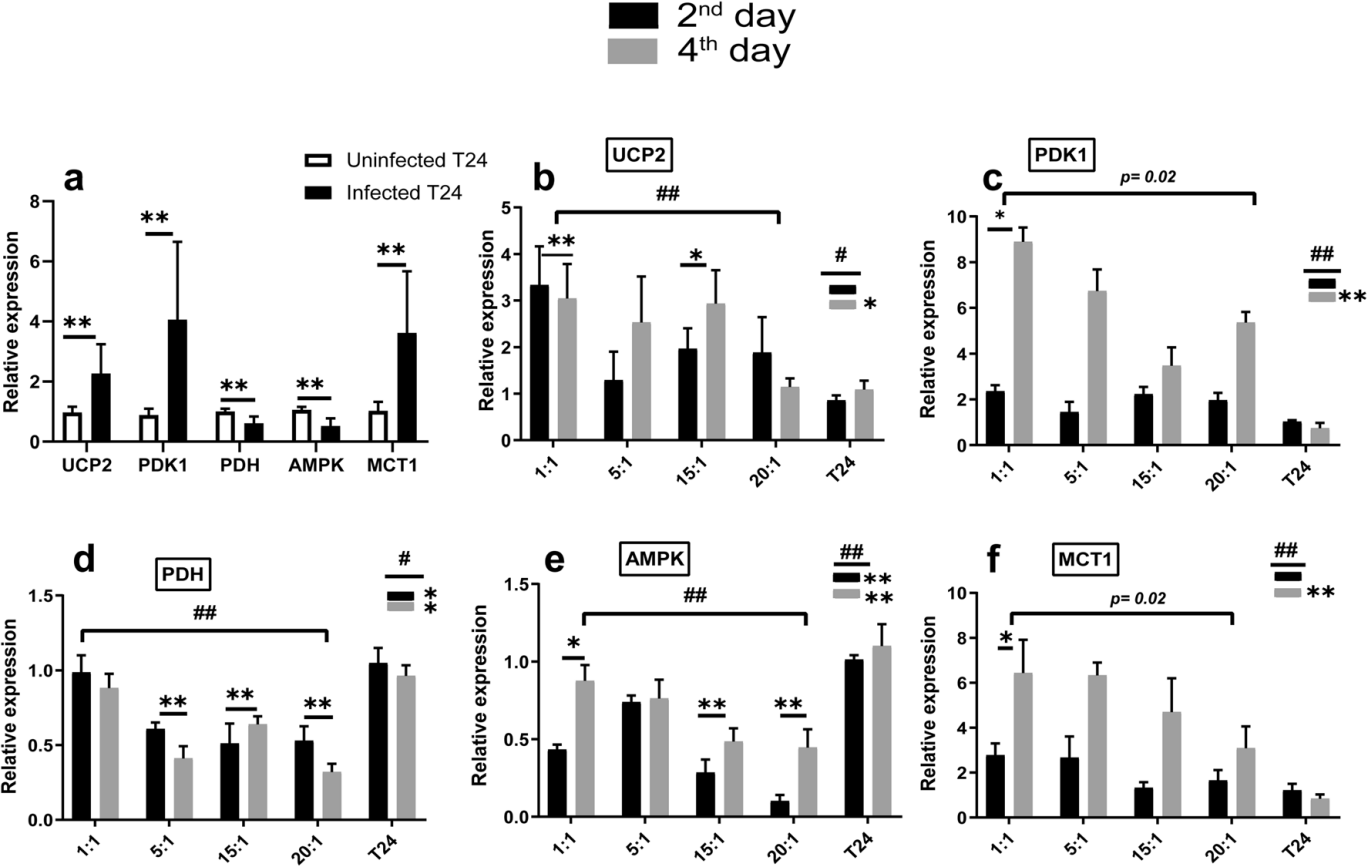
Effect of *E. coli* infection on relative gene expression of metabolic reprograming-related genes: (**a**) shows the pattern of UCP2, PDK1, PDH, AMPK and MCT1 expression in T24 cell line before and after *E. coli* infection. **Express the significance (*p* ≤ 0.001) obtained by independent t test. Infected T24 n = 24, control T24 n = 6. (**b**, **c**, **d**, **e**, **f**) show the UCP2, PDK1, PDH, AMPK and MCT1 expression in T24 cell line after *E. coli* infection at different MOI and durations, compared to uninfected T24 cells. #, ## express the significant difference (*p* ≤ 0.01) and (*p* ≤ 0.001) of gene expression with respect to the infection duration and MOI respectively, obtained by one-way ANOVA. *, **Refers to significant difference (*p* ≤ 0.01) and (*p* ≤ 0.001) obtained by Post Hoc Dunnett t (2-sided) for each MOI and each duration compared against the control T24 cells. 2nd day n = 12, 4th day n = 12, each MOI n = 6 and control n = 6.

Overall, the infected T24 cells recorded the highest mean expression levels of UCP2 (2.2 ± 0.9 folds), PDK1 (4 ± 2.5 folds), and MCT1 (3.6 ± 2 folds) relative to control (Fig. 5a), as well as the lowest mean expression level of PDH (0.6 ± 0.2 folds) and AMPK (0.5 ± 0.2 folds), relative to control (Fig. 5a). Regarding to bacterial stimulation, the UCP2, PDK1and MCT1 transcript levels increased significantly at *p* ≤ 0.001, while the PDH and AMPK transcript levels decreased significantly at *p* ≤ 0.001, when compared with the corresponding levels in uninfected T24 cells (Fig. 5a). At 4th day after *E. coli* stimulation, the highest expression levels were detected for UCP2, PDK1 and MCT1, with fold increase about (1.4 ± 0.4, 5.2 ± 0.7 and 4 ± 0.6), while PDH and AMPK transcription levels decreased down to (0.3 ± 0.1 and 0.4 ± 0.1) folds relative to controls. During the four days following the infection, with respect to MOI, the UCP2, PDH and AMPK levels have changed significantly at *p* < 0.001, while MCT1 and PDK1 levels have changed significantly at *p* < 0.05. Furthermore, with respect to infection duration, the PDK1, AMPK and MCT1 levels have changed significantly at *p* < 0.001, while UCP2 and PDH levels have changed significantly at *p* < 0.01. Post Hoc Dunnett t (2-sided) test was performed to confirm the significance at each MOI and duration separately compared to control (Fig. 5b–f). Thus, these findings demonstrate a comparable expression pattern of the glycolytic pathway-inducer genes and OXPHOS-related genes, which considered as metabolic alteration indicators.

## Discussion

Alteration of tumor microenvironment commands cancer development, here one model of that alteration will be discussed, which is the bacteria inhabiting tumors. Bučević Popović et al.^17^ has reported that microorganisms contribute up to 20% of human malignancies and has pointed out the probability of bacterial presence within bladder cancer patients. Therefore, understanding the mechanism by which bacteria influence tumor environment may facilitate solving the mystery of cancer progression and help therapy development. In current study, we screened the impact of in vitro bacterial infection of BC cell line (T24) on cellular trans-differentiation (EMT and stemness) as well as cellular metabolism.

The master characteristic of EMT is the activation of some transcription factors that lead to increase of mesenchymal-related proteins in advance of epithelial ones. CK19 is a subtype of cytokeratins that are the main structural protein forming intermediate filaments of epithelial cytoskeleton^19^. Therefore, downregulation of CK19 refers to lose in structural integrity of epithelial cell. In the other side, vimentin is the major cytoskeletal component of mesenchymal cells as it forms their intermediate filaments. It coexists with keratins in some metastatic cells which undergoing EMT. This reflects a phenotypic characteristic that engages to the aggressive and migratory behavior of epithelial tumors. Vimentin induces changes in cell shape, adhesion and motility during EMT, but the mechanism of that remains poorly understood. This study reveals that bacterial infection triggers EMT features such as reduction of epithelial marker (CK19) and upregulation of mesenchymal marker (vimentin). Reduction of epithelial markers have been highlighted in our previous research among infected bladder cancer patients less than those without infection^18^. This finding goes hand in hand with study performed by Leone et al.^20^ as it was reported *K*. *pneumoniae *ability to promote EMT-like programs in airway epithelial cells through direct interaction. Moreover, Song et al.^14^ indicated that—in oral squamous carcinoma cells- periodontal pathogens induce EMT through different pathways differ with bacterial types. In addition, EMT-induction in various organs including lungs and intestine was reported due to persistent chronic inflammation caused by microbial challenge^21^. That finding may be attributed to that bacterial invasion leads to transforming growth factorβ (TGFβ) modulation that induces signaling pathways that target downstream EMT transcriptional regulators^4,21,22^. A further example of the unbalanced microenvironment homeostasis is *Pseudomonas aeruginosa* chronic infection as it synergizes with the TGF-β1 to drive EMT to airway epithelial cells^20^.

EMT and CSCs engage a molecular network that potentially related to bladder cancer biology including tumor heterogeneity, transdifferentiation, and tumor progression^23^. EMT-related program is thought to act as a central driver of tumor development mechanisms, in particular CSCs^3^. It was pointed out through this study that *E. coli* stimulation implicated in increasing transcription levels of CD44. CD44 is a family of non-kinase transmembrane glycoproteins found on embryonic stem cells and on other cell types^24^. Hyaluronic acid (HA) is the main ligand for CD44, which is a constituent of the extracellular matrix (ECM) and it is expressed by stromal and cancer cells. Our results go hand in hand with the suggestion of CD44 has role in inflammation reduction and bacterial dissemination increase^25^. Previous study found that urothelial cells essentially express CD44 to facilitate *E. coli* infection and invasion of the urinary tract^26^. It illustrated that CD44 may serve as microorganisms binding site, so that *E. coli* could bind to HA adherent to CD44 on urothelial cells, which facilitates *E. coli* migration through epithelial cells. Moreover, van der Windt et al.^27^ reported that, CD44 absence influences host response and reduces bacterial dissemination^27^. From other prospect, CD44 is regarded as a molecular marker for CSC^24^. Bladder CSCs differ from other CSCs as they arise from bladder cancer cells at the late stages^24^. Hence, the resulted increase in CD44 either directly after bacterial stimulation, or due to EMT may refer to acquisition of stem cell-like properties. This is confirmed by our finding of upregulation of the three stemness markers SOX2, NANOG and OCT4. These transcription factors regulate self-renewal and maintenance in CSCs and have reported to be overexpressed in aggressive cancers, including muscle invasive BC^23,28^. Migita et al.^23^ positively correlated the increase in bladder cancer stemness and EMT. Our finding agrees, to some extent, to a previous study demonstrated that intestinal bacteria might activate a pathway, which in part orchestrates stemness of colon cancer^29^. In addition to Masaki et al.^30^ who reported gradually shut down of cell differentiation program and reprogram adult Schwann cells to stem cell-like cells due to *Mycobacterium leprae* infection^30^.

Efficient use and amount of the metabolites limit tumor growth, as they are spatially and temporally heterogeneous. To adapt this heterogeneity cancer cells shift their metabolism more toward glycolysis and away from oxidative metabolism^5^. Metabolic alterations also may allow cancer to adapt to environmental stressors and support the energy required for phenotypic changes like EMT and stemness and for rapid proliferation of cancer cells^31^. In the current study, evaluation of switching cancer cell metabolism from OXPHOS toward glycolysis, in course of bacterial infection, was firstly monitored through the stimulation of the oxidative stress. ROS have revealed a significant elevation due to bacterial stimulation in comparison to non-infected cancer cells which agrees with the previous studies that have reported the induction of ROS level due to bacteria^20,32^. The stimulation of the oxidative stress as well as metabolic alteration was also confirmed by the upregulation of a redox sensor protein UCP2. UCP2 (uncoupling proteins) is a mitochondrial inner membrane protein, which transports anion and regulates cell metabolism^10,33^. In fact, UCP2 is declined during early tumorigenesis stages to permit increase of ROS level and genomic instability. However, in the cancer development later stages it is provoked to induce anti-apoptotic mechanisms through ROS attenuation, causing cancer progression and therapy resistance^33^. Furthermore, UCP2 is considered as a glucose oxidation gatekeeper; it blocks pyruvate entrance to TCA cycle, leads to shift the cancer cell metabolism and preserve the Warburg effect^10^. Cordani et al.^33^ have also illustrated the role played by UCP2 to sustain the glycolytic pathway of pancreatic adenocarcinoma cells. This role is performed by transactivation of the gene encoding PDK1, which in turn downregulates PDH. PDK1, a key enzyme regulates glucose metabolism, attenuates ROS production and prevents hypoxia-induced apoptosis^34^. Zhang et al.^35^ has reported that PDK1 stimulates EMT program, resistance to cisplatin and increases ovarian cancer growth. In addition, it has been recognized as a gatekeeper of the Warburg effect^35^ through inhibition of PDH enzyme, which is a TCA cycle enzyme that is responsible for mitochondrial pyruvate conversion to acetyl-coA^7^. Hence, that may lead to aberration of mitochondrial oxidative metabolism, which explains our finding of downregulation of AMPK, a mitochondrial activity sensor. AMPK enzyme regulates cellular adaptation to energetic stress by engaging appropriate cellular responses to cope with metabolic perturbations. Upregulated AMPK activates mitophagy or mitochondrial biogenesis, while downregulated AMPK supports cell survival as it leads to stimulation of the Warburg effect^36^. However, there are conflicting reports about the role of AMPK signaling in EMT and cancer metastasis, which interpret that AMPK activation might have cell-type and context-specific effects^37^.

Another finding that supports the bacterial stimulation for metabolic switch as well as EMT is MCT1 increased level in cancer cells after infection. MCT1 is a proton-linked monocarboxylate transporter, which exports lactate to the ECM. ECM is a vital histological barrier that prevents cancer cells from metastasizing to a distant region. However, the acidic stress results from glycolysis, lactate production and acid accumulation on ECM enhance EMT by morphological alterations like cell compression and elongation^3^.

To sum up, with respect to our results, ROS levels are triggered in cancer cells because of bacteria that led to increase in UCP2 to adjust ROS levels. This enhances PDK1 to downregulate PDH in order to block OXPHOS in favor of glycolysis. That results in aberration in mitochondrial function and decrease of AMPK enzyme, while MCT1 elevates to be able to export the produced lactate to ECM. ECM now became more acidic and ready for morphological changes. This process occurs concomitantly with EMT as it provides the cell rapid ATP and energy required for transdifferentiation like EMT and stemness.

## Supplementary information

Supplementary Information

## Data Availability

The datasets generated during and/or analyzed during the current study are available from the corresponding author on reasonable request.
