# Supplementary material for: Escherichia coli foster bladder cancer cell line progression via epithelial mesenchymal transition, stemness and metabolic reprogramming
Source: Sci Rep. 2020 Oct 22;10:18024. doi: 10.1038/s41598-020-74390-5 (PMC7581527; doi:10.1038/s41598-020-74390-5)
Supplement: Supplementary file 1 — Supplementary Information [file 41598_2020_74390_MOESM1_ESM.pdf]

Title: *E coli* Foster Bladder Cancer Cell Line Progression via Epithelial Mesenchymal Transition, Stemness and Metabolic Reprogramming

Author list: Romaila Abd-El-Raouf[1, 2], Salama A. Ouf*[2], Mahmoud M. Gabr[1], Mahmoud M. Zakaria[1], Khaled F. El-Yasergy[2], Bedeir Ali-El-Dein[3]

Supplementary table S1: The results of biochemical tests for *E coli* isolate

Biochemical Details																	
2	APPA	-	3	ADO	-	4	PyrA	-	5	IARL	-	7	dCEL	-	9	BGAL	+
10	H2S	-	11	BNAG	-	12	AGLTp	-	13	dGLU	+	14	GGT	-	IS	OFF	+
17	BGLU	-	18	dMAL	+	19	dMAN	+	20	dMNE	+	21	BXYL	-	22	BAlap	-
23	ProA	-	26	LIP	-	27	PLE	-	29	TyrA	-	31	SURE	-	32	dSOR	+
33	SAC	+	34	dTAG	-	34	dTRE	+	36	CAT	-	37	)MN7	-	39	SXG	-
40	ILATk	+	41	AGLU	-	42	SUCT	+	43	NAGA	-	44	AGAL	+	45	PHOS	+
46	GlyA	-	47	ODC	-	48	LDC	+	53	IHISa	-	56	CMT	+	57	BGUR	+
58	O129R	+	59	GGAA	-	61	IMLTa	-	62	ELLM	-	64	ILATa	-			

Supplementary table S2: The results of antimicrobial susceptibility of *E coli* isolate

**Susceptibility Information**	**Analysis Time: 9.00 hours**			**Status Final**	
**Antibiotic**	**MIC**	**Interpretation**	**Antibiotic**	**MIC**	**Interpretation**
Ampicillin	**≥32**	**R**	Imipenem	**≤0.05**	**S**
Amoxicillin/ Clavulanic Acid	**≥32**	**R**	Amikacin	**4**	**S**
Ticarcillin	**≥128**	**R**	Gentamicin	**≤1**	**S**
Piperacillin/ Tazobactam	**≥128**	**R**	Tobramycin	**≥16**	**R**
Cefalotin	**≥64**	**R**	Nalidixic Acid	**≥32**	**R**
Cefoxitin	**≥64**	**R**	Ciprofloxacin	**≥4**	**R**
Cefotaxime	**≥64**	**R**	Ofloxacin	**≥8**	**R**
Ceftazidime	**≥64**	**R**	Nitrofurantoin	**≥512**	**R**
Ertapenem	**≥0.05**	**S**	Tfimethoprim/ Sulfamethoxazole	**≥320**	**R**

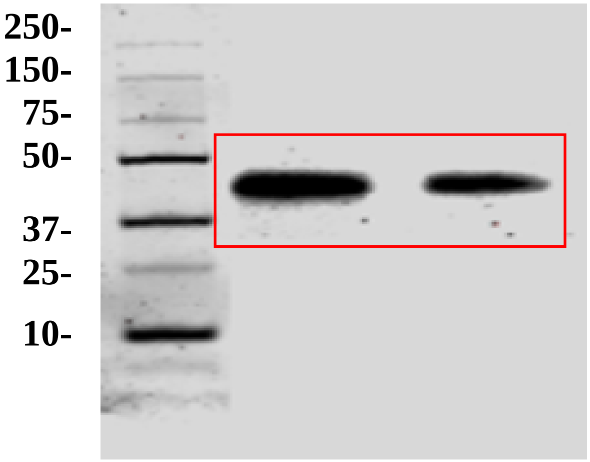

CK19 protein generated from western blot analysis.

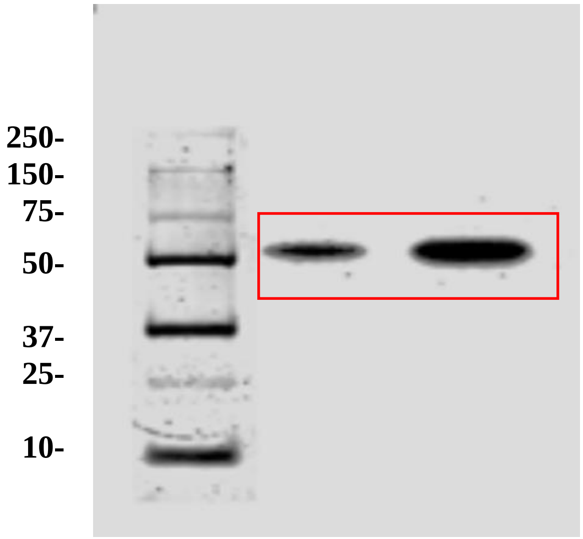

Vimentin protein generated from western blot analysis.

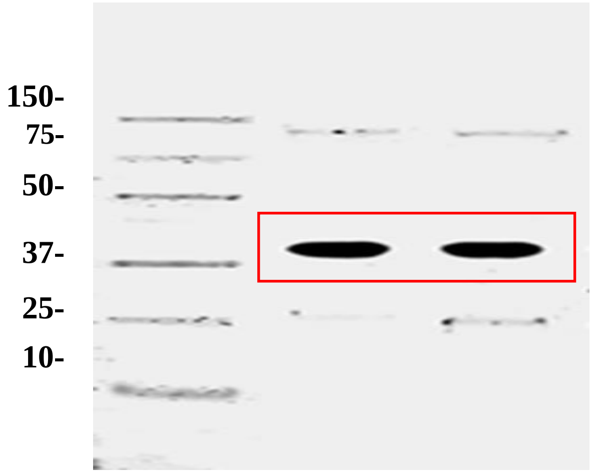

β-Actin protein generated from western blot analysis.

Supplementary Figure S2: The figure shows the full-length uncropped blots for CK19, vimentin and β-Actin western blots of Fig 2d displayed in the text results.
